# High Temperature Stable Anatase Phase Titanium Dioxide Films Synthesized by Mist Chemical Vapor Deposition

**DOI:** 10.3390/nano10050911

**Published:** 2020-05-09

**Authors:** Qiang Zhang, Chaoyang Li

**Affiliations:** 1 School of Systems Engineering, Kochi University of Technology, Kami, Kochi 782-8502, Japan; 216005z@gs.kochi-tech.ac.jp; 2 Center for Nanotechnology, Kochi University of Technology, Kami, Kochi 782-8502, Japan

**Keywords:** anatase, titanium dioxide, thin film, thermal stability, mist chemical vapor deposition

## Abstract

Pure anatase-phase titanium dioxide films stable up to high temperatures were successfully fabricated by the mist chemical vapor deposition method. A post-annealing treatment of the synthesized films was carried out in oxygen atmosphere in the temperature range from 600 to 1100 °C and no anatase to rutile transformation was observed up to 1000 °C. Based on the grazing incidence X-ray diffraction data, the average crystallite size of the titanium dioxide films increased gradually with increasing annealing temperature. The structural analysis revealed that the high thermal stability of the anatase phase can be attributed to the small crystallite size and a sheet-like grain structure. An incomplete anatase to rutile transformation was observed after annealing at 1100 °C.

## 1. Introduction

Titanium dioxide (TiO_2_) is one of the most promising semiconductor materials due to its unique properties such as a wide bandgap, abundance in nature, and high physical and chemical stability [1,2]. TiO_2_ has been extensively investigated for potential application in photocatalysts, photovoltaic devices and gas sensors [3]. Generally, TiO_2_ has three crystalline phases: anatase, rutile, and brookite. Compared with other phases of TiO_2_, anatase phase TiO_2_ has better photocatalytic and photovoltaic activity because of its larger bandgap and higher surface energy. However, anatase is the metastable phase and can easily transform to rutile phase, the most stable phase of TiO_2_, after high temperature heating. Reported research has shown that the anatase to rutile transformation occurs at temperatures between 450 and 850 °C, depending on fabrication method and precursors [4,5,6,7,8]. Various methods such as metal dopant and morphology control have been investigated to resist the anatase-rutile transformation [9,10]. However, until now, there was little report on successfully fabricating pure anatase TiO_2_ film stabilized at high temperature of over 900 °C. In order to apply anatase phase TiO_2_ for various environmental applications such as gas sensors, anti-microbial sanitary wares, and self-cleaning ceramic tiles, high temperature stable anatase TiO_2_ without phase transformation is essential [11,12]. Therefore, the development of a high temperature stable (above 1000 °C) anatase phase TiO_2_ is desirable.

The anatase to rutile transformation has been investigated by a number of groups [9,10,13,14,15,16,17,18,19,20,21,22]. According to the literature [9,10,13,14,15,16,17,18,19,20,21], the anatase to rutile transformation could be influenced by several factors including particle size, particle shape, and the presence of {112} facets. Related researches predicted that anatase became more stable than rutile when the particle size was smaller than the critical size (6.9~22.7 nm) [13,14]. Furthermore, it has been reported that rutile could easily nucleate at {112} twin interfaces during the anatase to rutile transformation [15,16,17,18,19]. As a result, the presence of {112} facets in anatase phase TiO_2_ will decrease the thermal stability of anatase phase TiO_2_.

According to our previous research, mist chemical vapor deposition (mist CVD) method was found to have specific advantages in terms of precise growth controllability, large area deposition and simplicity to synthesize TiO_2_ thin films [23,24]. In this research, the mist CVD method was used to synthesize TiO_2_ thin film, and the thermal stability of obtained TiO_2_ films was investigated.

## 2. Materials and Methods 

### 2.1. Fabrication of TiO_2_ Thin Films

TiO_2_ thin films with a thickness of 300 nm were deposited on quartz glass substrate (MITORIKA Co., Ltd., Mito, Japan) by mist CVD. The deposition condition of TiO_2_ films is shown in Table 1. A solution of precursor was prepared by dissolving titanium tetraisopropoxide (TTIP, purity > 95.0%, Wako Pure Chemical Industries, Ltd., Osaka, Japan) in ethanol (purity > 99.5%, Wako Pure Chemical Industries, Ltd., Osaka, Japan). The concentration of TTIP was 0.10 mol/L. The solution was ultrasonically atomized by ultrasonic transducers (2.4 MHz). Subsequently, the mist droplets of precursor were transferred to the reaction chamber using compressed air as a carrier gas and dilution gas, with flow rates controlled at 2.5 L/min and 4.5 L/min respectively. The substrate was set in the reaction chamber, which was heated and kept at 400 °C. 

### 2.2. Annealing Treatment

According to previous publications and TiO_2_ phase diagram, both the annealing ambient and pressure effect the temperature and speed of anatase to rutile phase transformation. However, the effect of annealing ambient on the phase transformation can be ignored because the variation of transformation temperature was less than 50 °C. Moreover, the effect of annealing ambient on the phase transformation was dependent on the structural properties of anatase TiO_2_. In gas sensor applications, the working conditions of gas sensors were oxidizing conditions (O_2_ or air) at room pressure (1 Bar). Therefore, in order to investigate the thermal stability, the as-deposited TiO_2_ films were calcined at a temperature in the range of 600–1100 °C in a pure oxygen ambient (1 Bar) for 1 h with a rapid thermal annealing furnace (RTA, ULVAC-RIKO, MILA-3000, Yokohama, Japan). The conditions of annealing treatment are shown in Table 2.

### 2.3. Characterization

The structural properties of TiO_2_ films were investigated by grazing incidence X-ray diffraction (GIXRD, ATX-G, Rigaku, Tokyo, Japan) with Cu Kα X-ray source (1.54184 Å) at a 0.35° incidence angle, Raman spectroscopy (LabRAM HR-800, Horiba Jobin Yvon, Longjumeau, France) with a 532.8 nm excitation laser, and X-ray photoelectron spectroscopy (XPS, AXIS-HS, Shimadzu/KRATOS, Kyoto, Japan) with Mg Kα X-ray source (1253.6 eV). The morphological properties of TiO_2_ films were evaluated by a field emission scanning electron microscope (FE-SEM, SU-8020, Hitachi, Tokyo, Japan). All measurements were carried out at room temperature. 

## 3. Results

**Figure 1:**
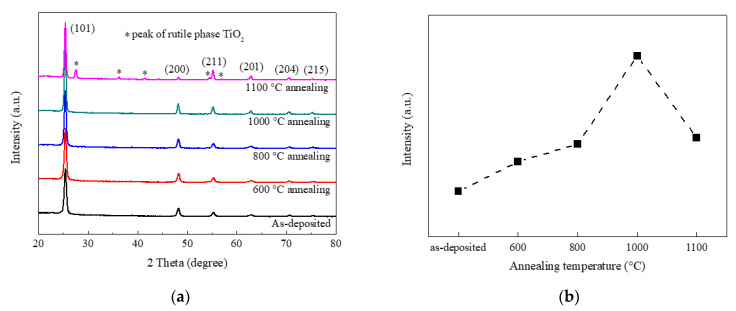
GIXRD results of as-deposited TiO_2_ films and TiO_2_ films after annealing. ((**a**) GIXRD patterns of TiO_2_ films; (**b**) (101) peak intensities of TiO_2_ films).

The GIXRD patterns of as-deposited TiO_2_ films and TiO_2_ films after annealing are shown in Figure 1a. Comparing as-deposited TiO_2_ films with TiO_2_ films calcined at 600, 800, and 1000 °C, it was found that all of the diffraction peaks corresponded with the diffractions from (101), (200), (211), (201), (204), and (215) crystal planes of the anatase phase TiO_2_. This suggested that pure anatase phase TiO_2_ thin films were obtained by mist CVD. No anatase to rutile transformation was observed after TiO_2_ films calcined at 600, 800, and 1000 °C. However, the TiO_2_ films calcined at 1100 °C showed a different GIXRD pattern. More peaks were observed at 2 theta of 27.57°, 36.24°, 41.44°, 54.52°, and 56.73° except the anatase phase peaks. Those peaks corresponded with rutile phase TiO_2_. This suggested that incomplete anatase to rutile transformation occurred under 1100 °C annealing.

Compared with other diffraction peaks for all samples, the (101) peak located at 2 theta of 25.45° was dominant with much higher intensity. The intensities of (101) peaks are shown in Figure 1b. Compared with as-deposited sample, the TiO_2_ films calcined at 600 °C showed higher (101) peak intensity. As the annealing temperature was increased from 600 to 1000 °C, (101) peak intensity gradually increased. When the annealing temperature was increased from 1000 °C to 1100 °C, the (101) peak intensity decreased due to the anatase to rutile transformation. 

It is well-known that the results of XRD, including peak intensity and the full width at half maximum (FWHM) value, can be used to calculate the grain size and the thickness of defective layer [25,26,27]. According to the Scherrer equation (Equation (1)), the (101) orientation crystallite size (L) of anatase TiO_2_ can be calculated using the FWHM value [25,26].

(1) $$L=\frac{K\lambda }{\beta \cos\theta }$$

where K is a constant related to crystallite shape (taken as 0.89 for anatase), *λ* the X-ray wavelength in nanometer (taken as 0.154184 nm in this research), *β* the FWHM value of diffraction peak in radians, and *θ* the diffraction angle. 

**Figure 2:**
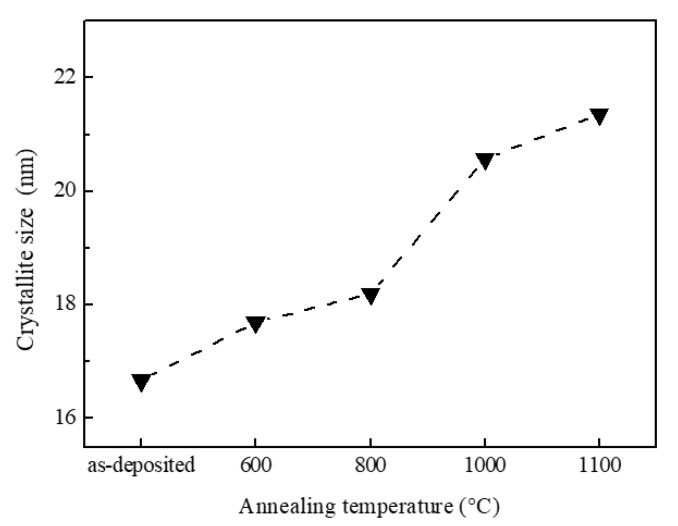
The (101) orientation crystallite sizes of as-deposited TiO_2_ films and TiO_2_ films after annealing.

Figure 2 shows the average crystallite size along the (101) orientation calculated from GIXRD data. The calculated crystallite size of as-deposited TiO_2_ films was around 16.7 nm. After annealing, the crystallite size of TiO_2_ films slightly increased. When the annealing temperature increased from 600 °C to 1100 °C, the crystallite size of anatase TiO_2_ increased from 17.7 nm to 21.4 nm gradually. The increase in crystallite size could be attributed to several factors, including the anatase to rutile transformation. Compared with the increase of anatase crystallite size caused by anatase to rutile transformation reported in other publications [20,28], the crystallite size increase of TiO_2_ synthesized by mist CVD was very gradual and slight. Therefore, we conclude that the increase in crystallite size was not caused by the phase transformation.

**Figure 3:**
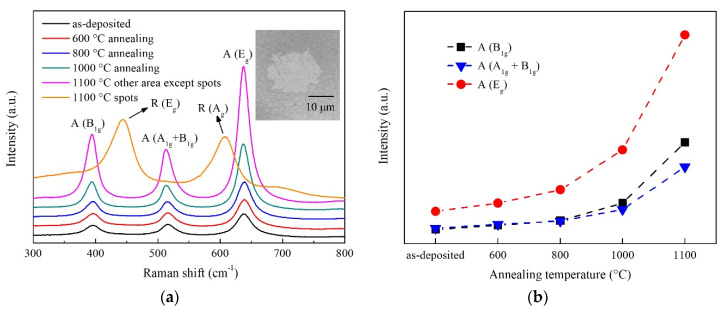
Raman results of as-deposited TiO_2_ films and TiO_2_ films after annealing. ((**a**) Raman spectra of TiO_2_ films; (**b**) Raman peak intensities of TiO_2_ films).

Raman spectra of as-deposited TiO_2_ films and TiO_2_ films after annealing are shown in Figure 3a. Three peaks were observed in the spectra of as-deposited TiO_2_ films and TiO_2_ films calcined at 600, 800, and 1000 °C. The peaks at around 395 cm^−1^ and 638 cm^−1^ corresponded with the B_1g_ mode and E_g_ mode of anatase phase TiO_2_ respectively [29,30]. The peaks at around 515 cm^−1^ corresponded with a doublet of the A_1g_ and B_1g_ modes of the anatase phase TiO_2_ [29,30]. All of the peaks corresponded with the anatase phase TiO_2_, which indicated the as-deposited TiO_2_ films were pure anatase phase and the anatase to rutile transformation did not occur after calcination at 600, 800, and 1000 °C. For TiO_2_ films calcined at 1100 °C, as shown in the inserted image, some spots with a diameter of around 20 μm were observed using the Raman system optical microscope. After checking these spots by Raman spectroscopy, two Raman peaks were observed and corresponded with rutile phase TiO_2_. Other areas were also measured and identified as anatase phase TiO_2_. This suggested that incomplete anatase-rutile transformation occurred during 1100 °C annealing. The results of Raman measurement were in agreement with those of the GIXRD measurement. Figure 3b shows the relationship between the intensities of anatase Raman peaks and annealing temperatures. Compared with as-deposited sample, the TiO_2_ films showed higher Raman peak intensities after annealing at different temperatures. As the annealing temperature was increased from 600 to 1100 °C, the intensities of all Raman peaks increased gradually.

**Figure 4:**
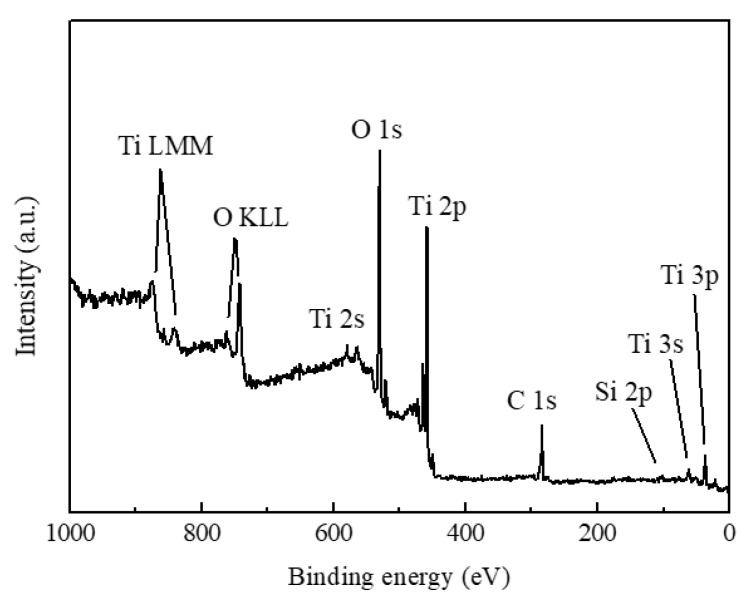
XPS survey spectrum of as-deposited TiO_2_ films.

Since the presence of dopants may strongly affect the thermal stability of anatase phase TiO_2_, the purity of as-deposited sample was investigated by XPS. Figure 4 shows the XPS survey spectrum of as-deposited TiO_2_ films. As shown in the XPS survey spectrum, several distinct peaks were observed and corresponded with various electron orbitals of titanium and oxygen [21,31,32,33,34]. The XPS peak at around 521.3 eV corresponded with the satellite peak of O 1s, which was attributed to Mg Kα 3. The Si 2p peak was observed at around 101 eV and attributed to the substrate (quartz glass). A C 1s peak at around 284.5 eV was observed due to environmental contamination. No peaks corresponding with other elements were observed, indicating that there were no obvious dopants in the as-deposited sample. Based on the results of XPS, the effects of impurities on the thermal stability of anatase phase TiO_2_ can be safely discounted.

**Figure 5:**
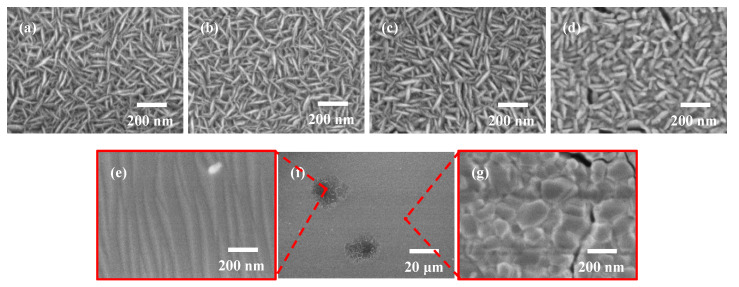
FE-SEM images of as-deposited TiO_2_ films (**a**) and TiO_2_ films calcined at different temperatures ((**b**) 600 °C; (**c**) 800 °C; (**d**) 1000 °C; (**e**–**g**) 1100 °C).

The top view FE-SEM images of as-deposited TiO_2_ films and TiO_2_ films after annealing are shown in Figure 5. It was confirmed that the as-deposited TiO_2_ film exhibited a uniform surface. As shown in Figure 5a, the intertwined TiO_2_ nanosheets were observed in as-deposited TiO_2_ film. Compared with as-deposited sample, the morphology of TiO_2_ films calcined at 600 °C and 800 °C including the length and thickness of sheet-like grains showed little change. As the annealing temperature was increased from 800 °C to 1000 °C, the thickness of TiO_2_ nanosheets increased slightly. However, the length of TiO_2_ nanosheets showed little variation. As shown in Figure 5f, the TiO_2_ films calcined at 1100 °C showed different morphology as other samples. Some spots with a diameter of around 20 μm were observed, which was similar to the image obtained by the optical microscope in Raman system. The detail of spots and other areas were checked and shown in Figure 5e,g respectively. According to Raman results, these spots were identified as rutile phase TiO_2_, and other areas were identified as anatase phase. However, the morphology of anatase phase area was different to other anatase phase samples. The grain size of anatase phase area was much bigger than other samples. This suggested that the incomplete anatase-rutile transformation occurred during 1100 °C annealing, which corresponds well to the results of the GIXRD and Raman measurement.

According to related research [6,22], if the anatase phase TiO_2_ is not thermodynamically stable at the critical temperature, the anatase to rutile transformation will occur immediately. At the beginning of phase transformation, the reaction rate is almost a constant. Accordingly, the rutile phase TiO_2_ can be detected after several minutes annealing, which has been proved by some publications [6,20]. In this research, the anatase phase TiO_2_ kept its sheet-like structure after one hour annealing at 1000 °C, and rutile phase TiO_2_ was not found by GIXRD and Raman measurement. Therefore, we can conclude that the anatase phase TiO_2_ films synthesized in this research showed high thermal stability up to 1000 °C.

According to the GIXRD results, the as-deposited TiO_2_ film showed a small crystallite size of around 16.7 nm. As mentioned in the introduction, anatase phase is thermodynamically more stable than rutile phase for such crystallite size. Moreover, the rutile transformation would be unlikely to occur if the crystallite size does not increase, which maybe a reason for the high thermal stability of TiO_2_ films. As mentioned in the introduction, the presence of {112} facets in anatase phase TiO_2_ will decrease the thermal stability of anatase phase TiO_2_. Therefore, a diffraction peak that corresponded with {112} facets was not observed in the GIXRD pattern of as-deposited sample, which also improved the thermal stability of TiO_2_ film.

Another reason for high temperature stabilized pure anatase TiO_2_ film could be the fabrication method. As annealing temperature was increased from 600 to 1000 °C, the morphology of TiO_2_ films including the length and thickness of sheet-like grains did not show much change. These unique sheet-like grains with fewer interfaces had a great influence on the anatase to rutile phase transformation. Fewer grain interfaces can suppress the phase transformation. The packing characteristics of the sheet-like TiO_2_ grains suppressed the interface nucleation of the rutile phase and significantly limited the phase transformation at a relatively high temperature. Moreover, the weak connections among sheet-like grains worked as barriers, which blocked anatase-rutile transformation. 

As the temperature increased to over 1000 °C, the interface and weak connections could not suppress the anatase to rutile transformation. The sheet-like grains were merged into a large grain with many voids.

## 4. Conclusions

Pure anatase phase TiO_2_ films with high thermal stability were successfully synthesized by mist CVD method. Sheet-like grains were observed in obtained TiO_2_ film. As calcined temperature was increased from 600 to 1000 °C, the TiO_2_ films did not show any obvious difference in terms of morphological and structural properties. The TiO_2_ films were in the pure anatase phase until the annealing temperature reached to 1000 °C. Anatase to rutile transformation of TiO_2_ film occurred at a temperature of 1100 °C. It was shown that the sheet-like grains, small crystallite size, and the absence of {112} facets might contribute to the high temperature stability of TiO_2_ film. Anatase TiO_2_ films with high temperature stability therefore demonstrate great potential for application in high temperature gas sensors.

## Figures and Tables

**Table 1:** Deposition condition of TiO_2_ films.

**Solute**	TTIP
**Solvent**	Ethanol
**Concentration (mol/L)**	0.10
**Deposition temperature (°C)**	400
**Carrier gas, flow rate (L/min)**	Compressed air, 2.5
**Dilution gas, flow rate (L/min)**	Compressed air, 4.5

**Table 2:** Conditions of annealing treatment.

**Ambient**	Pure Oxygen
**Pressure (bar)**	1
**Annealing time (h)**	1
**Annealing temperature (°C)**	600, 800, 1000, 1100
**Speed of warming up (°C/min)**	15
**Speed of cooling down (°C/min)**	10
